# Creating assessments as an active learning strategy: what are students’ perceptions? A mixed methods study

**DOI:** 10.1080/10872981.2019.1630239

**Published:** 2019-06-27

**Authors:** Josh B Kurtz, Michael A Lourie, Elizabeth E Holman, Karri L Grob, Seetha U Monrad

**Affiliations:** Department of Internal Medicine, Division of Rheumatology, University of Michigan Medical School, Ann Arbor, MI, USA

**Keywords:** Qualitative research methods, quantitative research methods, curriculum development/evaluation, active learning, multiple-choice questions

## Abstract

**Background**: Teaching students how to create assessments, such as those involving multiple-choice questions (MCQs), has the potential to be a useful active learning strategy. In order to optimize students’ learning, it is essential to understand how they engage with such activities.

**Objective**: To explore medical students’ perceptions of how completing rigorous MCQ training and subsequently writing MCQs affects their learning.

**Design**: In this mixed methods exploratory qualitative study, eighteen second-year medical students, trained in MCQ-writing best practices, collaboratively generated a question bank. Subsequently, the authors conducted focus groups with eight students to probe impressions of the process and the effect on learning. Responses partially informed a survey consisting of open-ended and Likert rating scale questions that the remaining ten students completed. Focus group and survey data from the eighteen participants were iteratively coded and categorized into themes related to perceptions of training and of collaborative MCQ writing.

**Results**: Medical students felt that training in MCQ construction affected their appreciation for MCQ examinations and their test-taking strategy. They perceived that writing MCQs required more problem-solving and content-integration compared to their preferred study strategies. Specifically, generating plausible distractors required the most critical reasoning to make subtle distinctions between diagnoses and treatments. Additionally, collaborating with other students was beneficial in providing exposure to different learning and question-writing approaches.

**Conclusions**: Completing MCQ-writing training increases appreciation for MCQ assessments. Writing MCQs requires medical students to make conceptual connections, distinguish between diagnostic and therapeutic options, and learn from colleagues, but requires extensive time and knowledge base.

## Introduction

Multiple choice questions (MCQs) remain a dominant method of assessing comprehension of medical knowledge in undergraduate medical education (UME), for both internally developed and external high-stakes assessments such as the USA Medical Licensing Examination (USMLE) Step 1 [1]. Students often report utilizing passive strategies to learn material for such assessments, such as re-reading notes [2]. Over the past several decades, medical educators have recognized the benefits of actively engaging students in order to optimize learning. Active learning is rooted in constructivist learning theories, which posit that learning occurs best when learners actively construct their own meaning rather than passively acquiring it; new knowledge builds on previously learned knowledge; and learners engage in authentic tasks [3,4]. This has driven a number of pedagogical changes in medical education, including strategies such as problem-based learning and team-based learning that promote active engagement with material.

Despite the ubiquitous use of MCQs in assessment, there are limited examples of medical students generating MCQs as a learning strategy [5–9]. MCQ-creation fulfills all of the key tenets of active learning described above. Generating information has been shown to improve students’ retention and comprehension of material both in the laboratory and classroom settings [10,11]. A review of intervention studies in reading comprehension that looked at comprehension of new material after being instructed on question-generation found effect sizes of 0.36 for standardized test performance and 0.86 for experimenter-generated test performance [12]. Of note, this review found that providing students with prompts and modelling for question generation resulted in better outcomes than less structured guidance.

Medical student involvement in MCQ-generation is associated with improvements in exam performance, although the rigor of the methodology used to assess this association has been variable. For example, first-year medical students who wrote MCQ distractors for a medical biochemistry course improved their post-test performance, consisting of the same questions they were given prior to the intervention [13]. Walsh et. al. demonstrated that students involved in question-writing performed significantly better on a subsequent institutional summative assessment, but did not control for baseline academic differences between those students who wrote MCQs and those who did not [8,9].These improvements in examination performance through involvement in MCQ-writing have also been shown in other healthcare professions training programs including nursing, pharmacy, and dentistry, but with similarly variable approaches ranging from associations between student ratings of peer-created MCQs and exam performance to true randomization with control group comparison [14–16].

Data on health professions students’ perceptions of MCQ-writing are similarly varied. While the majority of students involved in select question-writing activities have reported that they found it to be beneficial to their learning [6,14,17] others have reported no benefit [16,18] or disapproval of MCQ-writing as a method of learning [19]. Specific benefits reported by students involved in MCQ-writing include experiencing increased engagement with the learning process, increased confidence in taking examinations, and an alternative method to demonstrate their competence [5,20]. Reports on student perceptions of the utility of collaborative MCQ generation are equally divided; some studies report that students find great benefit from group work [7,14] while others suggest that MCQ-writing does little to encourage collaboration, or that the collaborative component is viewed unfavorably by students [21,22].

The majority of the above studies use quantitative survey data to understand student perceptions of MCQ-writing, and only one involving pharmacy students uses rigorous qualitative methodology [20]. Although quantitative analysis can provide a partial view into students’ view of question-writing, a rigorous qualitative approach is needed to provide essential data that may be missed by traditional quantitative methodology. Additionally, the extent to which students were trained to write MCQs varied widely, from no formal training received to holding in-person training sessions. Given that most successful studies examining MCQ-generation involve direct or detailed written instruction on MCQ-writing [23,24], studies involving formal training will likely provide more useful data.

A richer understanding of students’ perceptions of MCQ-writing would help us to understand how the process of question-writing influences students’ learning and more fully understand the strengths and limitations of question-writing as a potential learning strategy in UME. As such, we used an exploratory qualitative approach involving focus groups and an online survey to explore second-year medical students’ (M2s) perceptions of completing question-writing training and writing MCQs. Our primary research questions were: 1. How does formal instruction in MCQ-writing affect students’ perceptions of MCQ assessment? 2. How do medical students perceive collaborative MCQ writing to affect their own learning?

## Materials and methods

### Approach and study design

Our study used a sequential exploratory mixed-methods design [25] to account for two key issues. First, only a limited number of M2s would be able to participate in the study between their first and second years of medical school, and thus, given the limited study population, we felt a mixed-methods design would facilitate methodologic triangulation. Additionally, we wanted to both probe students’ perceptions through qualitative methods, and subsequently corroborate these opinions via quantitative methods. We chose an exploratory qualitative approach to inquiry, as we wished to gain a rich understanding of students’ experiences with the different components involved in question training and writing but were limited in our ability to use a fully grounded theory approach [26].

Our approach necessitates acknowledgment of our own perspectives and investment in the study and its participants. Two authors (JK and ML) are students in the same class as the study population; two (KG and BH) were members of the medical school’s evaluation and assessment unit; and one (SUM) directed the preclinical curriculum the students were in at the time of the study. Toward ensuring that our conclusions were not overly representative of one group, we intentionally structured each component of the study to include multiple stakeholders. JK, ML, BH and SUM were involved in the preparation of qualitative data collection; JK, ML and SUM participated in qualitative data analysis; JK, BH, and SUM participated in quantitative data analysis. All authors participated in integrating data toward achieving consensus on the most appropriate conclusions to be drawn.

### Context and sampling strategy

**Figure 1.**
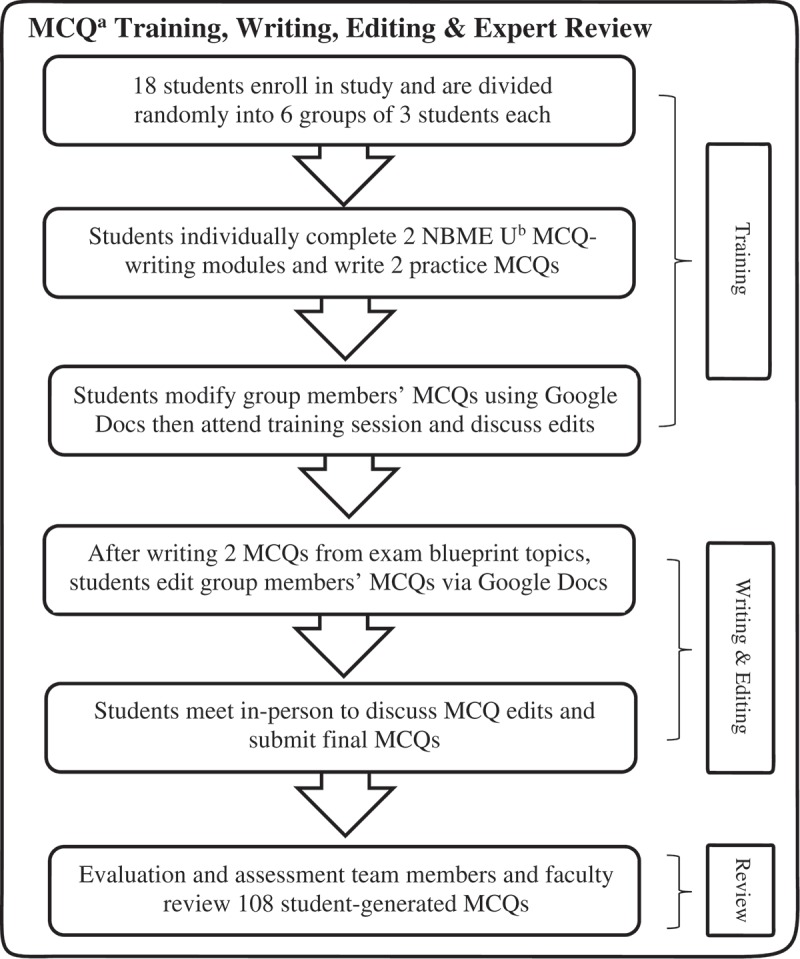
Training, writing and editing process for 18 second-year medical students involved in multiple choice question generation, University of Michigan Medical School, 2017.

We utilized a convenience sampling strategy of the University of Michigan Medical School (UMMS) M2s who participated in a summer project designed to generate a preclinical cardiology question bank for use by subsequent cohorts of medical students. All participants had taken the cardiology course, from which MCQs were to be created, nine months prior to the beginning of the study. To generate the study population, all 167 M2s received a recruitment email at the beginning of their 10-week summer between their first and second years. Response rate was limited by students’ geographic availability given other summer commitments. Students were offered up to $100 as an incentive for participation in and completion of all aspects of the project. The goal was to recruit twenty M2s in order to generate a substantial question bank. Of the twenty participants initially enrolled, eighteen completed the study. The question-writing training, writing, and editing that these 18 students completed is subsequently described and shown in Figure 1.

### Training in MCQ writing

All students first individually completed two online modules from NBME University (NBME U – nbmeuonline.com) on best practices for writing clinically-oriented MCQs (‘Structuring Multiple-Choice Items’ and ‘MCQ Flaws and How to Avoid Them’). Next, students were randomly assigned to three-member groups. Each student wrote one practice MCQ and suggested edits to their group members’ questions using Google Docs™. Subsequently, students attended an in-person MCQ-writing training session, wherein two of the authors (JK and KG) reviewed best practices. Students then discussed their suggested edits with group members. Informed consent was signed by all participants during the training session.

### Question bank creation

We created a two-dimensional 108-question exam blueprint [27] and assigned topics and question types accordingly (see Supplemental Digital Appendix 1). Students chose their own subtopics based on their review of course content. Next, they wrote two MCQs and suggested edits to their group members’ questions via Google Docs. To assist with question-writing, we provided students with modified NBME U Question Templates and Vignette Worksheets, as well as a modified UMMS MCQ-writing checklist (see Supplemental Digital Appendices 2–3). During the in-person editing sessions, students discussed their comments, modified their MCQs and re-submitted them. Each student completed this cycle of creating, editing, and collaboratively reviewing two MCQs three times, resulting in six MCQs generated per student. The final 108 edited MCQs underwent review by KG for MCQ-writing flaws and two cardiology faculty members for content accuracy.

### Data collection and analysis

**Figure 2.**
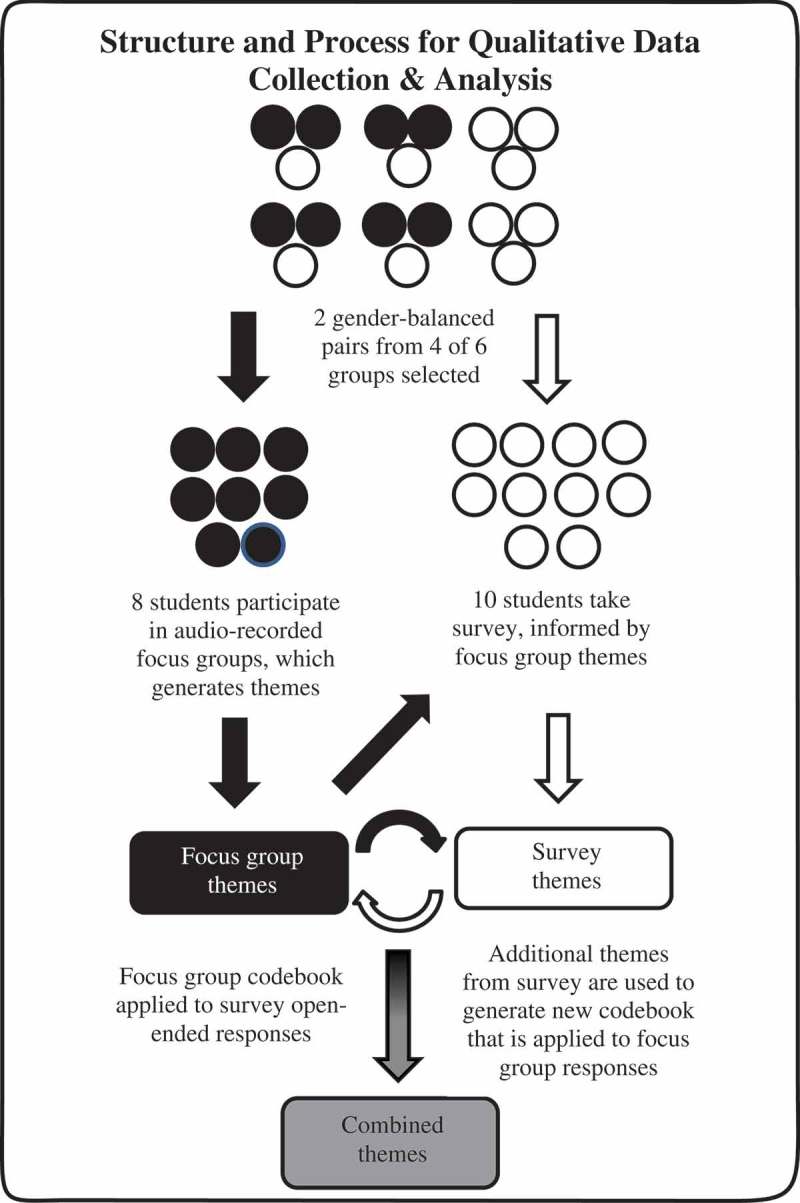
Process for qualitative analysis of focus group participants (n = 8) data and open-ended survey responses (n = 10) from 18 second-year medical students trained to write multiple choice questions, University of Michigan Medical School, 2017.

Our approach to data collection and analysis is outlined in Figure 2. After the last question-editing session, student pairs were chosen to participate in two gender-balanced focus groups, consisting of four students each. Students were selected in pairs to allow for discussions around group work, and pairs were gender-balanced to best represent the study population. Two authors (JK and ML) led, but did not themselves participate in, the one-hour focus groups, which they recorded and subsequently transcribed. Participants were asked nine open-ended questions (see Supplemental Digital Appendix 4), which were partially informed by discussions between JK and participants during the question-editing sessions. Specific probes were used to elicit elaboration when necessary.

We used thematic analysis to organize and describe the data, and an inductive approach to theme generation [28]. JK and ML read the focus group transcripts in an open coding process. Through constant comparison, coding categories were refined and updated iteratively. Both authors subsequently compared and challenged each other’s categorizations until they reached a consensus on categories that were best supported by the qualitative data. These common categories were used to create a code book, which JK and ML then used to re-code the data.

We used the categories generated from the focus groups to partially inform a survey, consisting of the same open-ended focus group questions and twelve multiple-part close-ended questions on a Likert scale from strongly disagree (1) to strongly agree (5). An expert in survey methodology reviewed the survey for content, clarity, and structure. We then asked two students to participate in concurrent cognitive interviewing using think aloud methodology and modified the survey accordingly to better align student understanding of question intent with our intended meaning [29]. The final survey was administered electronically via Qualtrics™ to the ten participants who had not participated in the focus groups. Two authors (SM and JK) used the previously generated code book to code the open-ended survey responses. Through an iterative process, relationships between and within focus group and survey response categories were drawn to generate themes. For the rating scale questions, we analyzed the data using Microsoft Excel 2016 (Microsoft Redmond, WA USA). Descriptive data were presented as a percentage.

### Ethics

The University of Michigan Institutional Review Board reviewed and approved the protocol (HUM 00129824).

## Results

Our findings are herein separated into student perceptions of the question-writing process as drawn from focus group and open-ended survey response data, and quantitative data drawn from the survey rating scale questions. Participants identified their gender as 50% male and 50% female, and ranged in age from 22 to 39 years of age with an average age of 26.

### Qualitative results

Our analysis revealed six themes regarding students’ perceptions of the process of question-writing: two regarding question-writing training and four regarding collaborative MCQ-writing. These themes are listed and described in Table 1.

**Table 1.** Major themes and their descriptions as derived from focus group and survey data from 18 second-year medical students trained to write multiple-choice questions, The University of Michigan Medical School, 2017.

Theme	Description
**Question-Writing Training**	
**Appreciation for the difficulty of MCQ-writing**	The technical challenges involved in creating high-quality MCQs
**Strategic test-taking**	How participating in MCQ-writing training changed students’ perceptions of using question-writing flaws to improve their test-taking strategy
**Question-Writing Process**	
**Integration of varying types and sources of information**	
*Concept integration*	MCQ-writing required integration of multiple content areas and intellectual concepts
*Information-source integration*	MCQ-writing required integration of information from multiple sources
**Differences in writing various parts of MCQs**	Student descriptions of the unique aspects of writing various components of the MCQ, including the stem, answer choices and explanation
**Community of practice in question-writing**	Acquisition of knowledge and skills from working and teaching other students
**Feasibility of MCQ-writing due to time and knowledge constraints**	
*Time constraints*	Student perceptions about the time-intensive nature of MCQ-writing as a learning approach
*Content knowledge constraints*	The challenge MCQ-writing posed for students with relatively limited medical knowledge in creating complex and authentic patient scenarios

#### Themes and representative quotes from question-writing training

##### Appreciation for the difficulty of MCQ-writing

Through participating in MCQ-writing training, students gained a deeper appreciation for the mechanics involved in creating a high quality MCQ that avoided common MCQ-writing flaws.

Survey Respondent (SR) 6: ‘I learned how hard it is to write good test questions! Not only do you have to have a thorough mastery of the material, but you have to match the information in the question with the expected knowledge of the students. Then you have to be able to clearly lead the learner to a specific answer without giving the answer away.’

SR3: ‘I learned the key pitfalls in designing questions that do not fairly assess a knowledge base (such as the confusion of having non-homogeneous answer choices), and how even though it is easy as a student to get frustrated on the receiving end of a MCQ with a major flaw, it is easy to make those mistakes when writing it.’

SR 1: ‘I also learned common pitfalls in writing questions and answer choices that may lead to confusion for test-takers or may inadvertently allude to an answer choice that would not require medical knowledge to solve.’

Students’ described the intricacies they deemed necessary to write MCQs appropriately tailored to the learner, suggesting that through receiving MCQ-writing training they experienced an increased awareness of the complexities of question-writing.

##### Strategic test-taking

Students also reported that completing MCQ-writing training altered their perceptions of how to strategically approach MCQ examinations, which they perceived to be potentially beneficial in taking future examinations.

Focus Group Respondent (FGR) 1 ‘Like looking at questions … when you take tests not all of the questions are going to be perfect and you can definitely use that to your advantage … through [question-writing training] I feel like you could be a better test-taker.’

SR 5: ‘It also taught me how to spot poor questions and the most likely right answer while test taking. I think that this experience will improve my test taking abilities.’

SR2: ‘The [training] modules helped me analyze multiple choice questions differently. I hadn’t previously looked at multiple-choice questions and considered how the question stem might lead me to a specific answer choice.’

Students perceived that learning to look for question-writing flaws through participating in MCQ-writing training made them more attuned to finding these flaws on future MCQ examinations, improving their own test-taking aptitude.

#### Themes and representative quotes from question-writing and editing

##### Integration of varying types and sources of information

Students described how question-writing required them to integrate multiple concepts, and to review and critically appraise information from multiple sources.

Focus Group Respondent (FGR) 1: ‘ … not only do you have to review the specific thing you have to write the question about but you also have to review why all the other answer choices are wrong.’

FGR 2: ‘ … I wasn’t sure if I was making a patient that sounded like something else, so I would look up a little more detail about the patient just to make sure that what we were given in lecture fully painted the picture.’

FGR 2: ‘The whole idea of an illness script and that like you are hitting on that whole idea of what presents similarly, what presents differently when you are writing your multiple choice question or thinking about it. I think that’s the difference between just doing the exercise and like studying the concepts more broadly.’

After identifying that there are subtle differences that distinguish diagnoses or treatment plans, students sought multiple resources to determine how to most accurately represent their chosen subtopic. Students also reported that they found authentically representing a patient with a given diagnosis to be a particular challenge; they addressed this difficulty through utilizing multiple resources to understand the broader picture.

##### Differences in writing various parts of MCQs

Students perceived writing certain parts of their MCQs to be more challenging than others. Writing the answer choices appeared to be particularly challenging, yet rewarding, because it required students to make subtle distinctions they had not previously considered.

SR 1: ‘I was surprised to find that it was sometimes difficult to come up with several reasonable answer choices, as I had to find answers that were plausible in the scenario but objectively incorrect.’

FGR 3: ‘I think the most valuable thing for me was coming up with the answer choices … to come up with good and tricky answer choices I needed to think through what are all the ways that if I read this quickly or wasn’t really thinking critically or jumping to like initial assumptions how would I answer this question. And that kind of working backwards helps you identify like what are the critical pieces of information that you would need to know about this disease process to be able to like effectively answer this type of question.’

FGR 3: ‘ … trying to come up with [answer choices] to some extent [made me] start thinking through differentials and how you prioritize things on a differential.’

Students discussed how writing answer choices presented a unique challenge compared to writing other components of the MCQ. By contrasting similarities and differences between the correct answer and the distractors, students were forced to think about the essential distinguishing characteristics of specific diagnoses and use clinical reasoning to create a differential diagnosis for their hypothetical scenario.

##### Community of practice in question-writing

Through the in-person editing sessions, students described the impact of working in groups. Students appreciated the opportunity to understand how their peers approached problem-solving and question-writing. They also felt they benefited from teaching others.

SR 6: ‘It was helpful to gain an understanding of how different question writers displayed different writing trends, despite attempting to write similarly structured questions.’

SR 7: ‘I think the most important part of this to me was the in-person discussion. It was really interesting to sit with the other people in my group and discuss how we approached writing our questions. Despite the similar subjects that we were writing about, we each had our own way of going through the writing process and our own style that was reflected in our questions.’

SR 2: ‘By reviewing each other’s questions, we reviewed content, and I think better understood the concepts by explaining them to one another.’

Through working in groups and reviewing other students’ MCQs, participants described a two-fold benefit. First, students felt that they learned the material in a different way by hearing information explained differently. Second, students gained an appreciation for different approaches to question construction and how they could use that to improve their own questions.

##### Feasibility of MCQ-writing due to time and knowledge constraints

In addition to the benefits of writing MCQs, students also voiced concerns regarding the amount of time and extensive knowledge base needed to write MCQs.

SR 4: ‘[Writing MCQs] is very different [than my preferred study strategies.] However, I still do not think it is the best method for me, at least. It is too time consuming for the amount of knowledge gain I can achieve.’

FGR 2: ‘ … part of the thing I was like in a battle with myself when I was writing these questions because I don’t know a whole lot about medicine yet, and like the context all these patients are put in like is it actually realistic? Does it mean something?’

SR 2: ‘Not knowing the content and needing to take the time to review it well enough to feel confident that my question was sound was the most challenging for me.’

Students expressed that they were concerned that the intensive time burden and amount of content knowledge required to write MCQs might limit its utility as a learning tool in the future. Given the subtle distinctions between certain diagnoses, students had to spend an extensive amount of time to research and write authentic patient scenarios, which they felt may not be the most efficient way to learn material presented in high volumes.

### Quantitative results

We herein report the results from the Likert scale questions from the 10 students who took the survey informed by the focus group responses. Students perceived completing question-writing training (mean = 3.7 ± 0.5), question-writing (mean = 3.9 ± 0.3), and question editing (mean = 3.8 ± 0.6) to be beneficial to their learning. In terms of question-writing training, students agreed that learning how to write MCQs changed their strategy for approaching MCQs (mean = 3.8 ± 1.0). Students neither agreed nor disagreed that completing question-writing training increased their confidence in taking MCQ examinations (mean = 3.2 ± 0.8). Similarly, students neither agreed nor disagreed that MCQ-writing training should be incorporated into the medical school curriculum (mean = 3.1 ± 1.1). Regarding the act of question-writing, students agreed that writing MCQs made them critically analyze differences between diagnoses (mean = 4.2 ± 0.6). However, students reported that they spent 50.5 minutes on average (mean = 50.5 ± 26.3) writing each MCQ and they neither agreed nor disagreed that question-writing was an efficient way to review cardiology material (mean = 2.9 ± 1.1). For question-editing, students agreed that participating in editing improved the quality of their MCQs (mean = 4.0 ± 0.7) and provided a beneficial exposure to practice MCQs (mean 4.10 ± 0.3). Students spent an average of 17.4 minutes (mean = 17.4 ± 8.7) reviewing and suggesting edits to their group members’ MCQs prior to the in-person editing sessions.

Students were asked to assess which part of writing MCQs made them think the most critically (Table 2). The majority of students (60%, n = 10) perceived that writing the answer choices required them to think the most critically. Students were asked to compare their preferred study strategies (re-reading lecture slides, re-writing notes, and reviewing flashcards) to MCQ writing, as shown in Table 3. Students felt that writing MCQs required much more time and slightly more integration of concepts and information sources, in addition to differentiation between diagnoses and problem solving.

**Table 2.** Second-year medical students’ (N = 10) perceptions of which part of the multiple-choice question made them think the most critically during the writing process, as reported by percentage of total survey responses, University of Michigan Medical School, 2017.

Question Part	Percent (%)
The answer choices	60%
The explanation	20%
The question vignette	10%
The question itself	10%
All parts made me think equally critically	0%
No parts made me think critically	0%

**Table 3.** Survey data of second-year medical students’ (N = 10) perceptions of how much of the following was required to write multiple-choice questions, compared to their preferred study strategies from much less (1) to much more (5), by mean and standard deviation (SD), University of Michigan Medical School, 2017.

Prompt	Mean	SD
Time	4.90	0.32
Problem solving	4.30	0.92
Integration of multiple concepts within a single course	4.10	0.74
Integration of information from multiple sources	4.00	1.05
Differentiation between diagnoses	4.00	0.82

## Discussion

This study was developed based on the principles of active learning. While involving medical students in active learning strategies such as question-writing is likely to improve retention of information, it is important to consider student perceptions of these activities in order to optimize their efficacy. Our results suggest that through completing question-writing training, students deepened their appreciation for the challenges involved in creating a well-written MCQ and enhanced their understanding of how to use MCQ-writing flaws to their advantage in taking MCQ examinations; participating in MCQ-writing training did not, however, increase students’ confidence in taking future MCQ examinations. In terms of question-writing itself, students perceived that it requires integration of information, subtle differentiation between diagnoses, and an extensive amount of knowledge. Students perceived that writing the answer choices and explanations forced them to think the most critically. They also felt that collaborative question-writing provided beneficial exposure to the different ways other students approached problem solving. However, an important tension with the positive attributes of question-writing was the extensive amount of time and content knowledge required to generate questions. Students also reported hesitation in incorporating MCQ-writing as a learning strategy in the medical school curriculum.

The findings we report here add to the growing body of knowledge surrounding health professions student involvement in question-writing. While some studies have used survey methodology to examine student perceptions of question-writing, few of these have supplemented this information with qualitative data, and none with the rigorous methodology that we utilized. Additionally, the extent of question-writing training in previous reports is highly varied with no reports drawing distinctions between student perceptions of question-writing training and the question-writing process itself.

Regarding previous survey reports of health professions students’ perceptions of MCQ-writing, first-year dental students reported that question-writing made them critically analyze information and justify their answer choices [14] and first-year pharmacy students felt that question-writing resulted in great time and depth of engagement [20]. Our findings build upon these results by demonstrating that MCQ-writing required students to draw connections across concepts and forced critical assessment of subtle differences between diagnoses. Further, it has not been previously reported that students perceived writing the answer choices to require the most critical thinking compared to the other components of MCQs. This finding is important, both in considering how to create more efficient question-writing activities for medical students and how to compensate for lack of clinical experience in generating plausible vignette-based stems.

Unlike other studies involving learners in question-writing, we required students to complete rigorous training in MCQ-writing both through online and in-person sessions. Students reported that their appreciation for well-written MCQs grew and their perceived approaches to MCQs were altered as a result of this study, which has not been previously reported. Our finding that students perceived question-writing to benefit their learning stands in contrast to previous reports which suggest that the minority of medical students find question-writing to be beneficial as a learning strategy [16,18,30]. This discrepancy may be explained in part due to the in-depth training medical students received on how to write MCQs in our study, improving understanding and appreciation for the writing process. It is important to note that although students reported benefits to their learning, they still demonstrated hesitation to incorporate question-writing in UME. This is likely due to the intensive time and knowledge burden required to write questions that we heard from students, and which has been previously reported [18,20].

**Figure 3.**
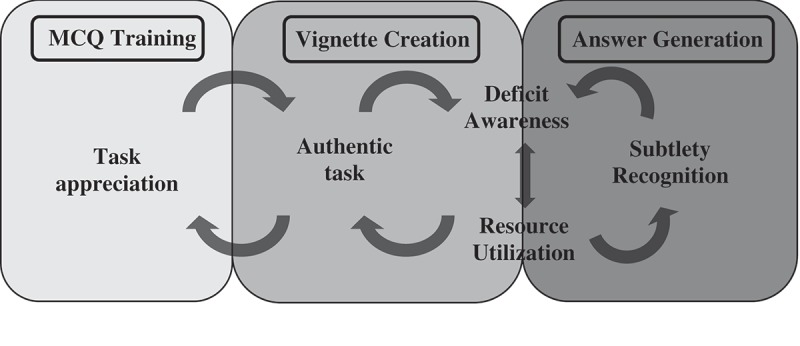
Conceptual framework for how collaborative MCQ-generation impacts medical student-learning as derived from focus group and survey data from 18 second-year medical students trained to write multiple-choice questions, The University of Michigan Medical School, 2017.

Based on our results, and building on existing literature, we propose a preliminary model for how engaging in collaborative training and question-writing impacts medical student learning as shown in Figure 3. First, through engaging in question-writing training, medical students gain a deep understanding of how MCQs are structured and designed, and thus an appreciation for this assessment modality beyond what occurs by simply answering MCQs. Developing patient vignettes for the MCQs provides authenticity to the task, as students strive to generate realistic clinical situations. Additionally, another level of authenticity arises from creating an assessment format that students engage with regularly throughout their training. The acquisition and integration of knowledge needed to generate authentic scenarios and plausible diagnoses resulted in students becoming acutely aware of their own knowledge deficits. In order to overcome these deficits, students utilize multiple resources (including their peers) and continue on this cycle until they deem their vignettes acceptable. In particular, students found their peers to be particularly valuable resources for assessing their understanding of content and approaches to learning and assessment. In writing answer choices for MCQs, medical students experience an additional layer of recognition of subtlety between diagnoses or treatment options that forces critical appraisal of options. This process makes them further aware of knowledge deficits, requiring further resource utilization.

Our findings suggest a number of important considerations for those interested in incorporating MCQ-writing as a learning strategy in health-professions curricula. First, offering students in-depth question-writing training may provide the unique benefit of altering their perception of and approach to answering MCQs. Second, students should be given ample time to write questions, as they perceive question-writing to require substantially more time than their preferred study strategies. Given the relatively significant time investment and independent work required for question-creation, this active learning approach may be best suited for medical school curricula which utilize problem-based learning and in which students are regularly expected to do independent pre-work prior to meeting. Alternatively, it may be appropriate to place the most emphasis on writing the answer choices and explanations instead of the question stem, as students perceived this part of question-writing to be the most beneficial; and writing plausible clinical vignettes the most challenging. Lastly, although some students may perceive collaborative question development and editing to be time intensive and difficult given relatively limited medical knowledge, it may provide the unique benefit of exposing students to the different ways by which students approach question-writing and problem solving.

The generalizability of the findings from this study are limited by a number of factors. Medical school curricula vary widely in their design, and it is possible that these findings are unique to students in this particular systems-based one-year pre-clinical curriculum. However, given that our findings are congruent with previous reports of health professions students finding MCQ-writing to be time intensive and require critical thinking skills, we feel this is unlikely [14,20]. Although the open-ended survey responses corroborated the focus group findings, because the interviewers were classmates of the participants, it is possible that the participants were less willing to share negative perspectives during the focus groups potentially limiting the generalizability of these findings [31]. In a focus group setting, it is also possible that the views expressed as a group differ from the personal viewpoints expressed in individual interviews [32,33]. For the quantitative survey data, the generalizability is limited by our small sample size, which minimized the utility of performing traditional calculations of statistical significance. Given that the qualitative data was used to inform the survey design, our data collection process and results are best characterized qualitative-dominant [34–37]. The quantitative findings are therefore best interpreted in coordination with the qualitative data. Finally, despite our use of cognitive interviewing and expert review to provide validity evidence, the survey has not been previously utilized or validated otherwise.

In conclusion, medical students perceive that writing MCQs requires more integration of material, problem-solving, and time compared to their preferred study strategies. Specifically, students perceive that writing the answer choices requires the most critical reasoning and forces them to make subtle distinctions between diagnoses. Additionally, students felt that receiving training on how to write MCQs affected their approach to taking multiple-choice examinations and that working with other students gave them a beneficial exposure to different question-writing approaches. Specific to question-writing, it is important to give students ample time to write questions, emphasize the potential benefits of exposure to different styles of question-writing and item-writing flaws, and have students spend the most time writing the portion of the question which they perceive to be the most beneficial. By incorporating the student perspective around active learning strategies, which may be unfamiliar and time intensive, these activities may be more positively received and perceived as more beneficial to students. Lastly, while pragmatic and logistic restrictions may necessitate an abbreviated approach to engaging learners in assessment creation, incorporating the key elements of our theoretical model for question-creation may be used to optimize learning experiences involving assessment generation.

## Data Availability

The data that support the findings of this study are openly available in Mendeley Datasets at http://dx.doi.org/10.17632/2r5w9t73y9.1.
