# Supplementary material for: Creating assessments as an active learning strategy: what are students’ perceptions? A mixed methods study
Source: Med Educ Online. 2019 Jun 27;24(1):1630239. doi: 10.1080/10872981.2019.1630239 (PMC6610530; doi:10.1080/10872981.2019.1630239)
Supplement: Supplemental Material [file ZMEO_A_1630239_SM1452.zip › 1630239_supplemenatry/supplemenatry.docx]

Appendices

**Supplemental Digital Appendix 1:**

**Exam blueprint for 108 multiple-choice questions generated by 18 second-year medical students, The University of Michigan Medical School, 2017.**

| Primary Framework: CV System | Secondary Framework: Domain | | | | |
| --- | --- | --- | --- | --- | --- |
|  |  |  |  |  |  |
| Lecture Topic | Diagnosis | Pathophysiology or Mechanism | Treatment | Basic Science/Physiology | Total |
| Cardiovascular Physiology | 0 | 0 | 0 | 14 | 14 |
| Conduction and EKG | 2 | 0 | 0 | 3 | 5 |
| Cardiovascular Pharmacology | 0 | 7 | 3 | 2 | 12 |
| Hypertension | 1 | 2 | 2 | 0 | 5 |
| Valvular Heart Disease | 4 | 0 | 3 | 0 | 7 |
| Acute Coronary Syndrome | 3 | 1 | 2 | 1 | 7 |
| Arrhythmias | 3 | 5 | 2 | 3 | 13 |
| Heart Failure | 3 | 4 | 2 | 1 | 10 |
| Peripheral Vascular Disease | 2 | 2 | 2 | 0 | 6 |
| Cardiomyopathy | 1 | 1 | 1 | 0 | 3 |
| Cardiovascular Infections | 2 | 0 | 1 | 0 | 3 |
| Pericardium and Pericardial Disease | 2 | 1 | 1 | 0 | 4 |
| Coronary Artery Disease | 1 | 1 | 2 | 0 | 4 |
| Approach to Chest Pain | 3 | 0 | 0 | 0 | 3 |
| Embryology and Fetal Circulation | 0 | 0 | 0 | 3 | 3 |
| Congenital Heart Disease | 1 | 2 | 3 | 0 | 6 |
| Pathology | 2 | 1 | 0 | 0 | 3 |
| Total | 30 | 27 | 24 | 27 | 108 |

**Supplemental Digital Appendix 2:**

**Modified NBME University (*adapted from nbmeuonline.com)* multiple-choice question templates as used by 18 second-year medical students trained to write multiple-choice questions, The University of Michigan Medical School, 2017.**

**Basic Science/Physiology Template**

The Basic Sciences Template can be used to structure a number of questions focusing on physiology or basic science concepts. The Lead-ins for these questions tend to be more variable, but examples are included below to guide your question creation.

| **Basic Science/Physiology Template** | **Sample Basic Science/Physiology Question** | | |
| --- | --- | --- | --- |
| **Question stem (patient vignette):**  Describe a patient with a problem.  **Example Lead-ins: Variable**  The oral hydration formula most likely promotes sodium absorption via the gut by allowing co-transport with which of the following?  Which of the following mechanisms help maintain the patient’s core temperature during the period following her rescue?  In this patient, PTH induces which of the following processes to cause hypercalcemia?  **Options:**  List of treatment options  Description of mechanism of action |  | A 39-year-old woman comes to the physician for a follow-up examination because she recently was diagnosed with  hypertension. Her blood pressure is 156/100 mm Hg. Physical examination shows no other abnormalities. Serum studies show normal findings. A 24-hour urine collection shows three times the normal excretion of epinephrine and metanephrine.  The excessive epinephrine production in this  patient is most likely caused by which of the following cell types?  (A) Chromaffin  (B) Juxtaglomerular  (C) Zona fasciculata  (D) Zona glomerulosa  (E) Zona reticularis |  |

**Supplemental Digital Appendix 3:**

**Multiple-choice questions writing checklist as used by 18 second-year medical students trained to write multiple-choice questions, The University of Michigan Medical School, 2017.**

Item Stems

- Stems present information in the context of a clinical or experimental vignette.
- Stems avoid using negative wording like NOT or EXCEPT.
- Stems can be answered without seeing the item responses (“cover-the-options” rule).
- Stems are formatted in the standard NBME style (use Vignette Worksheet):

1. Patient’s age and gender

2. Site of care

3. Chief complaint/presenting complaint (include nature of onset)

4. Duration of symptoms (include changing nature of symptoms)

5. Pertinent history (HPI, personal, medications, family)

6. Examination findings

7. Results of diagnostic studies

8. Response to initial treatment

9. Lead-in

Item Responses

- There are 3-8 possible responses.
- There is a single best answer (avoids “B and C” types of answer choices).
- Response options do not include “all of the above” or “none of the above.”
- Response options are similar in length.
- Response options include a single dimension (all diagnostic tests, medications, diseases, etc.).
- Response options are grammatically consistent with the question stem.
- Response options follow a logical order (alphabetical, numbered list, chronological, etc).
- Response options avoid repeating words or phrases that are included in the questions stem.
- Response options avoid ambiguous terms and phrases like *associated with, is important, is useful for, may, usually, or rarely.*
- Response options avoid extreme terms like *never, only, nothing, and alone*.
- Distractors do not overlap (i.e. 1-3, 3-5, 5-7).

Solutions

- An explanation is given for each answer choice that clearly indicates why it is correct or incorrect
- The explanation for each answer choice starts with “Answer A (B, C, D, etc) is correct (or incorrect).”

**Supplemental Digital Appendix 4:**

**Semi-structured interview guide used during focus groups with eight second-year medical students trained to write multiple-choice questions, The University of Michigan Medical School, 2017.**

| **Semi-structured interview guide** |
| --- |
| - Why did you volunteer to be involved in this study? |
| - What did you learn from completing training about how to write multiple choice questions? |
| - What did you learn from writing multiple choice questions? |
| - What did you learn from reviewing other students’ questions? |
| - How did your experience in learning about and writing MCQs affect your motivation to study? |
| - How would you say question writing compares to other study strategies you have used? |
| - What was most challenging about the process of question writing? |
| - What recommendations would you offer to students using MCQ generation as a learning tool? |
| - We are interested in understanding how being involved in question writing has impacted your learning. Is there anything else you would like us to know? |
